# Detection of relevant amounts of cow’s milk protein in non-pre-packed bakery products sold as cow’s milk free

**DOI:** 10.1186/2045-7022-5-S3-O8

**Published:** 2015-03-30

**Authors:** Valérie Trendelenburg, Nadeschda Enzian, Johanna Bellach, Sabine Schnadt, Bodo Niggemann, Kirsten Beyer

**Affiliations:** 1 Charité Universitätsmedizin Berlin, Berlin, Germany; 2 German Allergy and Asthma Association, Mönchengla, Germany

## Background

Currently there is no mandatory labelling of allergens for non-pre-packed foods. Therefore, when buying products sold loose, consumers with food allergy rely on voluntary information provided orally by the staff or in a written form in the salesroom. The aim of this study was to evaluate whether the staff in bakeries is able to give advice regarding a safe product choice for food allergic consumers and to what extent bakery products, which are sold as “cow’s milk-free” by the staff, contain cow’s milk protein.

## Methods

Staff of 50 bakeries in four different neighbourhoods in Berlin was interviewed regarding selling non-pre-packed foods to customers with food allergy. Bakery products being recommended as “cow’s milk-free” by the staff were bought and cow’s milk protein levels were measured with ELISA ((*RIDASCREEN^®^FAST Milk* (*RBiopharm*)).

## Results

In 30/50 bakeries (60%) the staff reported that they serve customers with food allergy at least once a month, in 12/50 bakeries (24%) at least once a week. Most of the staff (42/50, 84%) stated that they are able to advise food allergic consumers regarding a safe product choice. Altogether 73 “cow’s milk-free” products were sold in 44/50 bakeries to the study personal. Cow’s milk protein could be detected in almost half of the bakery products (43%), every fifth product (19.2%) contained > 3 mg cow’s milk protein.

## Conclusion

Most of the staff in Berlin bakeries felt confident in advising costumers with food allergy. However cow’s milk was detectable in a great number of bakery products which was sold by the staff as “cow’s milk-free”. In some products the quantity of cow’s milk protein exceeded an amount where approximately 10% of cow’s milk allergic children show clinical relevant symptoms.

